# NADPH-dependent ROS accumulation contributes to the impaired osteogenic differentiation of periodontal ligament stem cells under high glucose conditions

**DOI:** 10.3389/fendo.2023.1152845

**Published:** 2023-06-07

**Authors:** Yi-lin Zhang, Ying An, Li-Juan Sun, Hong-Lei Qu, Xuan Li, Xiao-Tao He, Rui-Xin Wu, Fa-Ming Chen, Bei-Min Tian, Yuan Yin

**Affiliations:** State Key Laboratory of Military Stomatology & National Clinical Research Center for Oral Diseases & Shaanxi Engineering Research Center for Dental Materials and Advanced Manufacture, Department of Periodontology, School of Stomatology, Fourth Military Medical University, Xi’an, China

**Keywords:** high glucose, osteogenic differentiation, ROS, NADPH, periodontal tissue regeneration

## Abstract

Diabetes mellitus is an established risk factor for periodontal disease that can aggravate the severity of periodontal inflammation and accelerate periodontal destruction. The chronic high glucose condition is a hallmark of diabetes-related pathogenesis, and has been demonstrated to impair the osteogenic differentiation of periodontal ligament stem cells (PDLSCs), leading to delayed recovery of periodontal defects in diabetic patients. Reactive oxygen species (ROS) are small molecules that can influence cell fate determination and the direction of cell differentiation. Although excessive accumulation of ROS has been found to be associated with high glucose-induced cell damage, the underlying mechanisms remain unclear. Nicotinamide adenine dinucleotide phosphate (NADPH) is an important electron donor and functions as a critical ROS scavenger in antioxidant systems. It has been identified as a key mediator of various biological processes, including energy metabolism and cell differentiation. However, whether NADPH is involved in the dysregulation of ROS and further compromise of PDLSC osteogenic differentiation under high glucose conditions is still not known. In the present study, we found that PDLSCs incubated under high glucose conditions showed impaired osteogenic differentiation, excessive ROS accumulation and increased NADPH production. Furthermore, after inhibiting the synthesis of NADPH, the osteogenic differentiation of PDLSCs was significantly enhanced, accompanied by reduced cellular ROS accumulation. Our findings demonstrated the crucial role of NADPH in regulating cellular osteogenic differentiation under high glucose conditions and suggested a new target for rescuing high glucose-induced cell dysfunction and promoting tissue regeneration in the future.

## 1 Introduction

Periodontitis is a microbially triggered inflammatory disease, characterized by destruction of teeth supporting structures (including gingiva, periodontal ligament (PDL) and alveolar bone), and ultimately leads to tooth loss if left untreated (1). Many studies have demonstrated that periodontal disease is closely associated with a wide range of systemic diseases, particularly Diabetes mellitus (2, 3). It is revealed that Diabetes mellitus, especially type 2 Diabetes mellitus, can exacerbate the prevalence and severity of periodontal diseases and delay the repair of periodontal defects. Worse yet, patients with diabetes exhibited more severe destruction of the periodontium than those without diabetes (4, 5). In this context, the chronic high glucose environment is considered as an important contributing factor to diabetic complications and diabetes-induced cell dysfunction (6). For example, stem cells isolated from diabetic rats showed increased apoptosis and decreased migration ability (7, 8). Although periodontal ligament stem cells (PDLSCs) have been recognized as the most promising candidate for the treatment of periodontitis and repair of periodontal defects (9–12), our previous studies also revealed that the osteogenic differentiation of PDLSCs was significantly impaired under high glucose conditions *in vitro (* 13). Hence, exploring the mechanisms underlying high glucose-induced PDLSC dysfunction is an essential prerequisite for stem cell-based periodontal treatment in diabetic patients.

The exact mechanisms involved are still mysterious, but studies have revealed that the high glucose-caused cell dysfunction may be associated with inhibition of Glo1/AGE/RAGE axis (14), PI3K/Akt pathway (15), activation of MAPK pathway (13), excessive generation of reactive oxygen species (ROS) (16) and so on. Among the mechanisms studied, ROS, small molecules derived from oxygen and participate in regulating cell fate and cell proliferation and differentiation (17, 18), has been recognized to be the most important contributor, and would be a potential target for periodontal treatment (19–21). Generally, increased ROS accumulation was found to be responsible for the reduced osteogenic differentiation potential of stem cells (22–24). Vice versa, under pathological circumstances, such as aging, inflammation and metabolic disorders, stem cells tend to differentiate into adipocytes, instead of osteoblast (25–27), which is consistent with the presence of increased ROS accumulation in stem cells during adipogenic differentiation (28). Evidence also suggested that compared to those under normal conditions, cells under high glucose conditions exhibited higher intracellular ROS levels, and in turn the increased ROS accumulation can accelerate the inflammasome activation and/or mitochondrial malfunction, leading to severe cell apoptosis and dysfunction (29, 30). Given the above findings, ROS accumulation plays an essential role in high glucose-induced cell dysfunction, but the exact mechanisms underlying high glucose-caused ROS accumulation in PDLSCs remain further study.

Nicotinamide adenine dinucleotide phosphate (NADPH) is an essential electron donor that functions as a ROS scavenger in cellular antioxidation systems (31). NADPH can promote the dispose of peroxides, such as H_2_O_2_, by binding to catalase (a key H_2_O_2_-disposing enzyme), and/or decrease ROS generation *via* activating glutathione reductase (an important enzyme of cellular antioxidant system) (32–34). In addition, NADPH can also scavenge cellular ROS by promoting the production of thioredoxin (an important antioxidant) (35). However, seemingly paradoxically, NADPH can be used as a substrate to activate NADPH oxidases (NOXs) and participate in the generation of ROS (31). Interestingly, it is reported that the increased cellular ROS levels could be indirectly cause by NADPH-induced NOXs reactivation under a high glucose environment (19). Despite increased NADPH levels have been found in functionally impaired cells derived from diabetic rats (19, 36), the exact role of NADPH, the relationship between ROS and NADPH and their influences on the osteogenic differentiation potential of PDLSCs under high glucose conditions remain poorly understood.

In the present study, we established high glucose conditions to mimic diabetes-related environment *in vitro* according to previous study (13), and observed the effects of high glucose conditions on the osteogenic differentiation potential of PDLSCs. Furthermore, the levels of intracellular ROS and NADPH were measured, and the effects of cellular NADPH on PDLSC under high glucose conditions were further explored. Our findings provide a new insight into the mechanisms underlying high glucose-induced PDLSC dysfunction and might provide a potential therapeutic targets of periodontal therapy for diabetic patients.

## 2 Materials and methods

### 2.1 Isolation and purification of PDLSCs

Human PDLSCs were isolated from healthy and intact third molars according to methods described in our previous studies (10, 13). All donors (n = 6, aged 18-28 years) have signed a written informed consent for research purposes before donation, and subsequent studies were approved by the Ethics Committee of the School of Stomatology, Fourth Military Medical University, Xi’an, Shaanxi, China. Briefly, extracted teeth were washed using cold phosphate-buffered saline (PBS; Gibco, New York, USA) and the gingival tissues were excluded thoroughly. Then, the PDL tissues were scraped from the middle third of the root surface, cut into small pieces and digested using Type I collagenase (Sigma-Aldrich, St. Louis, USA) at 37 °C for 60 min. Subsequently, the PDL tissues were resuspended in Dulbecco’s modified Eagle’s medium (DMEM) supplemented with 10% (v/v) fetal bovine serum (FBS), 1% penicillin and streptomycin (all from Gibco). The medium was refreshed every 2 days until the primary cells migrated from the tissues. Finally, the limiting dilution technique was used for the purification of PDLSCs, and cells at passage 2-5 (P2-5) were used in subsequent studies. To eliminate individual differences from different donors, cells from same donors were cryopreserved and thawed at the same time points for subsequent experiments.

### 2.2 Identification of PDLSCs

#### 2.2.1 Colony-forming unit assay

The colony-forming capacity of PDLSCs was assessed by a CFU assay. In brief, PDLSCs were seeded in 100-mm-diameter culture dishes (Invitrogen, Carlsbad, CA, USA) at a density of 8 × 10^2^ cells/dish for 12 days. The cell colonies were then stained with 0.1% toluidine blue (Sigma-Aldrich) and photographed using an inverted microscope (Olympus Optical, Tokyo, Japan).

#### 2.2.2 Cell Counting Kit-8 assay

The proliferation ability of PDLSCs was measured using the CCK-8 assay. PDLSCs were seeded in 96-well culture plates (Invitrogen) at a density of 8 × 10^2^ cells/well. During an 8-day culture, culture medium were removed, cells at each well were washed and incubated with 200 μL medium added with 20 μL CCK-8 reagent (Invitrogen) at 37 °C for 2 h at a proscribed time point every day. Then, cellular proliferative capacity of PDLSCs were detected with a microplate reader (TECAN, Männedorf, Switzerland) at 450 nm absorbance.

#### 2.2.3 Alizarin Red staining

The osteogenic differentiation potential of PDLSCs was detected using Alizarin Red staining. In brief, PDLSCs were seeded in 6-well plates (Invitrogen) at a density of 2 × 10^5^ cells/well and cultured until 60–70% confluence. Cells were then incubated with osteogenic induction medium: complete DMEM supplemented with 50 μg/mL vitamin C, 10 nmol/L dexamethasone and 10 mmol/L β-glycerophosphate (all from Sigma-Aldrich). The medium was refreshed every 2 days. After a 21-day osteogenic induction, cells were fixed with 4% paraformaldehyde (Invitrogen), and the mineralized nodules were stained with Alizarin Red solution (Cyagen, Guangzhou, China) and then photographed with an inverted microscope.

#### 2.2.4 Oil Red O staining

The adipogenic differentiation potential of PDLSCs was detected using Oil Red O staining. Briefly, PDLSCs were seeded in 6-well plates at a density of 2 × 10^5^ cells/well and cultured until 80–90% confluence. Cells were then incubated with adipogenic induction medium (Cyagen). The medium was refreshed every 2 days. After a 21-day adipogenic induction, cells were fixed with 4% paraformaldehyde, and the lipid droplets were stained with Oil Red O solution (Cyagen) and then photographed with an inverted microscope.

#### 2.2.5 Alcian Blue staining

The chondrogenic differentiation potential of PDLSCs was detected using Alcian Blue staining. In brief, PDLSCs were collected in 15-mL centrifuge tubes (Invitrogen) at a density of 2 × 10^5^ cells/tube and then cultured with chondrogenic induction medium (Cyagen) until those cells aggregated and formed a suspending cell cluster. The medium was refreshed every 2 days. After a 21-day chondrogenic induction, the cell clusters were fixed with 4% paraformaldehyde, and the chondrogenic differentiation potential of PDLSCs was evaluated using Alcian Blue solution (Cyagen) according to previous studies (13, 37).

#### 2.2.6 Flow cytometry analysis

The immunophenotypes of PDLSCs were identified using Flow cytometry analysis. According to the International Society for Cell & Gene Therapy (ISCT) definition of mesenchymal stem cells (MSCs) and our previous studies (13, 38–40), PDLSCs were incubated with monoclonal antibodies against human CD11b, CD19, CD31, CD34, CD45, CD73, CD90, CD105, CD146 and HLA-DR (all from eBioscience, San Diego, USA). Cells incubated with PBS served as the blank controls. The immunophenotypes of PDLSCs were then assessed using a Beckman Coulter Epics AL cytometer (Beckman Counter, Fullerton, USA).

### 2.3 Cell treatment

To identify the effects of high glucose conditions on the osteogenic differentiation potential of PDLSCs, cells were incubated with osteogenic induction medium (refer to 2.2.3 for details) prepared with complete DMEM (5 mmol/L glucose; referred as the Ctrl group) or high glucose DMEM (25 mmol/L glucose; referred as the HG group). To reduce NADPH production, cells were preincubated with glucose-6-phosphate dehydrogenase (G6PDH) siRNA (GenePharma, Shanghai, China) for 48 h or 100 nmol/L specific G6PDH inhibitor 6-aminonicotinamide (6-AN; MedChemExpress, Monmouth Junction, NJ, USA). To investigate the role of NADPH in cellular osteogenic differentiation, PDLSCs were treated with differentiation concentrations of NADPH (0-500 nmol/L, MedChemExpress) under normal glucose conditions.

### 2.4 Alkaline phosphatase staining and ALP activity measurement

ALP staining and ALP activity measurement was performed to measure the osteogenic potential of PDLSCs after a 7-day osteogenic induction. PDLSCs were fixed using 4% paraformaldehyde for 45 min and then stained with a BCIP/NBT ALP Color Development kit (Beyotime, Shanghai, China). The ALP staining-positive cells were observed and photographed with an inverted microscope. ALP activity measurement was performed using an ALP assay kit (Nanjing Jiancheng Bioengineering Institute, Nanjing, China) according to the manufacturer’s instructions. Briefly, the supernatant was added to a 96-well plate, and 50 μL of reagent I and II was added to the wells. After incubation at 37 °C for 15 min, 150 μL reagent III was added to the wells and cellular ALP activity of PDLSCs were detected with a microplate reader at 560 nm absorbance.

### 2.5 Measurement of intracellular ROS levels

The levels of intracellular ROS were determined with measuring the oxidative conversion of cytomembrane permeable non-fluorescent probe 2’, 7’-dichlorofluorescein-diacetate (DCFH-DA, Beyotime) to fluorescent dichlorofluorescein (DCF) by flow cytometry analysis. PDLSCs were seeded in 96-well plates at a density of 1 × 10^3^ cells/well and incubated with 10 μmol/L DCFH-DA at 37°C in the dark for 30 min. The converted fluorescent DCF was observed and photographed using an Olympus FV1000 confocal laser scanning microscope (CLSM; Olympus, Tokyo, Japan) and analyzed using FV10-ASW4.2 software. The relative fluorescence intensity of DCF was recorded using a Beckman Coulter Epics AL cytometer, with an excitation wavelength of 488 nm and emission wavelength of 525 nm.

### 2.6 Measurement of intracellular NADP, NADPH, NADP^+^ levels and NADPH/NADP^+^ ratio

The NADP, NADPH, NADP^+^ levels and NADPH/NADP^+^ ratio were measured using an NADP^+^/NADPH Assay Kit with WST-8 (Beyotime) according to the manufacturer’s instructions. Briefly, PDLSCs were seeded in 6-well plates at a density of 2 × 10^5^ cells/well for 48 h and then lysed with NADP^+^ /NADPH extraction buffer. Each lysed sample was collected and divided into two parts: one part was heated at 60°C for 30 min to deplete NADP^+^ (heated sample, only NADPH left), while another part was left on ice (unheated sample, containing both NADP^+^ and NADPH). Then, absorbance values were measured at 450 nm was detected with a microplate reader. Intracellular NADP, NADPH, NADP^+^ levels and the NADPH/NADP^+^ ratio were calculated using the following formula: Total NADP = intensity of unheated sample, Total NADPH = intensity of heated sample, Total NADP^+^ = intensity of unheated sample – intensity of heated sample, NADPH/NADP^+^ ratio = (intensity of heated sample)/(intensity of unheated sample – intensity of heated sample).

### 2.7 Quantitative real-time polymerase chain reaction

QRT-PCR was conducted to measure the mRNA expression levels. In brief, total RNA was extracted from cells with TRIzol reagent (Invitrogen) and then reverse-transcribed to cDNA using Evo M-MLV RT Premix (Takara, Shiga, Japan). qRT-PCR was performed using the SYBR Green Premix Pro Taq HS qPCR kit (Tli RNaseH Plus; TaKaRa), and the results were analyzed with a CFX96 Real-time RT-PCR system (Bio-Rad, Hercules, CA, USA). β-Actin was used as the house-keeping gene, and the expression of target genes was calculated using the 2^−ΔΔCT^ method. The primer sequences used for qRT-PCR were as follows: *β-Actin*: sense 5’-TGGCACCCAGCACAATGAA-3’ and antisense 5’-TGGCACCCAGCACAATGAA-3’; *ALP*: sense 5’-AACATCAGGGACATTGACGTG-3’ and antisense 5’-GTATCTCGGTTTGAAGCTCTTCC-3’; *RUNX2*: sense 5’-TGGTTACTGTCATGGCGGGTA-3’ and antisense 5’-TCTCAGATCGTTGAACCTTGCTA-3’; *BMP2*: sense 5’-ACCCGCTGTCTTCTAGCGT-3’ and antisense 5’-TTTCAGGCCGAACATGCTGAG-3’; *OCN*: sense 5’-CCCAGGCGCTACCTGTATCAA-3’ and antisense 5’-GGTCAGCCAACTCGTCACAGTC-3’; *G6PDH*: sense 5’-GACCTACGGCAACAGATACAAGA-3’ and antisense 5’-GCAGTGGGGTGAAAATACGC-3’.

### 2.8 Western blot analysis

Western blot analysis was performed as previously described (13). Briefly, cells were lysed by RIPA lysis buffer supplemented with protease and phosphatase inhibitors (all from Beyotime), and the protein concentrations were measured using a bicinchoninic acid (BCA) assay kit (Beyotime). Then, the protein samples were separated by sodium dodecyl sulfate–polyacrylamide gel electrophoresis (SDS-PAGE; Beyotime) and transferred to PVDF membranes (Millipore, Billerica, MA, USA). After being blocked with 5% nonfat milk (Beyotime) at room temperature for 90 min, the membranes were incubated with primary antibodies at 4°C overnight and then incubated with secondary antibodies (1:20000; goat anti-rabbit IgG, SAB, L3012; goat anti-mouse IgG, SAB, L3032). The blots were visualized using an enhanced chemiluminescence substrate (Millipore), and protein bands were analyzed with ImageJ software. β-Actin was used as the housekeeping protein for internal normalization. The following primary antibodies were used for western blot analysis: β-Actin (1:2000; Proteintech, 20536), ALP (1:10000; Abcam, ab108337), RUNX2 (1:1000; CST, #12556), BMP2 (1:1000; Abcam, ab214821), and OCN (1:1000; Santa Cruz; Sc-390877).

### 2.9 siRNA transfection

The method for siRNA transfection was performed to regulate the expression of G6PDH. In brief, PDLSCs were seeded in 6-well plates at a density of 2 × 10^5^ cells/well and cultured until 60–70% confluence, then those cells were transfected with siRNA targeting G6PDH (si-G6PDH) or negative control siRNA (NC) in the presence of transfection reagents (GenePharma). After a 48-h transfection, the enzymatic activity and mRNA level of G6PDH was detected. Validated si-G6PDH and NC were packaged by GenePharma. The sense sequence of NC was 5’-UUCUCCGAACGUGUCACGUTT-3’, and the antisense sequence was 5’-ACGUGACACGUUCGGAGAATT-3’.

### 2.10 Statistical analysis

All data are presented as the mean ± standard deviation (SD) of at least three independent experiments. GraphPad Prism 9 software was used for statistical analysis: unpaired two tailed Student’s *t* test was conducted for comparisons between two unpaired groups and one-way analysis of variance followed by Tukey’s multiple comparisons tests, Sidak’s multiple comparisons tests or Dunnett’s multiple comparisons were performed for comparing more than two groups. Statistical significance was expressed as ^*^ *p* < 0.05, ^**^ *p* < 0.01 or ^***^ *p* < 0.001.

## 3 Results

### 3.1 High glucose conditions compromised the osteogenic differentiation potential of PDLSCs

Human PDLSCs were successfully isolated and purified from PDL tissues (**Figure S1A**). The stem cell characteristics of these cells were measured using CFU assay, CCK-8 assay, analyses of multidifferentiation potential and flow cytometric analysis. These PDLSCs were able to generate new colonies (**Figure S1B**), multiply *in vitro* (**Figure S1C**), differentiated into multilineage cells (**Figure S1D**) and were also positive for mesenchymal-associated markers (**Figure S1E**), indicating that the isolated and purified cells were stem cells and can be used for further experiments.

**Figure 1:**
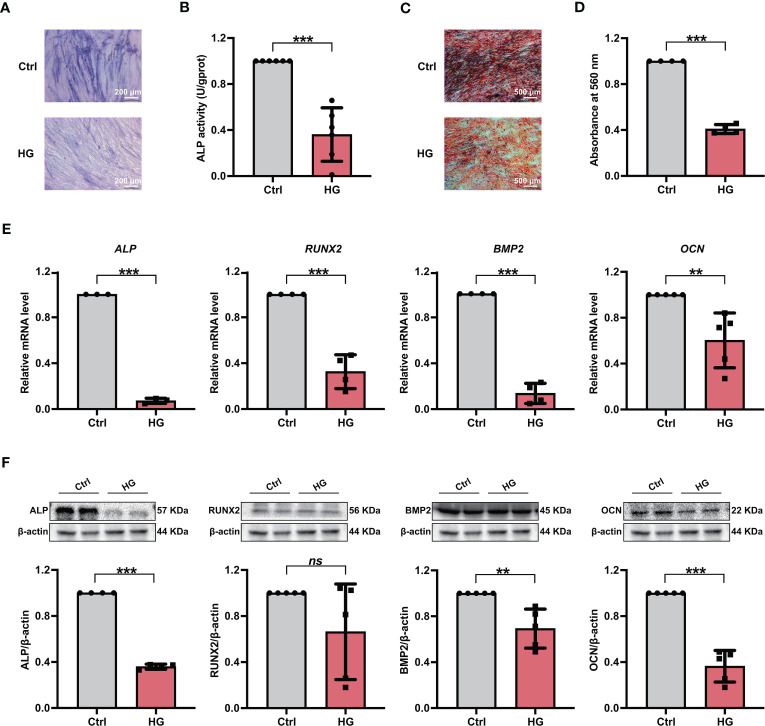
Incubation of PDLSCs under high glucose conditions led to impaired osteogenic differentiation. **(A)** ALP staining of PDLSCs under normal (Ctrl) or high glucose (HG) conditions following a 7-day osteogenic induction (scan bar = 200 μm). **(B)** ALP activity assay of PDLSCs under normal (Ctrl) or high glucose (HG) conditions following a 7-day osteogenic induction. **(C)** Alizarin Red staining of PDLSCs under normal (Ctrl) or high glucose (HG) conditions following a 21-day osteogenic induction (scan bar = 500 μm). **(D)** Quantitative analysis of mineralized nodules formed by PDLSCs under normal (Ctrl) or high glucose (HG) conditions following a 21-day osteogenic induction. **(E)** Expression levels of osteogenesis-related genes (*ALP*, *RUNX2*, *BMP2* and *OCN*) in PDLSCs under normal (Ctrl) or high glucose (HG) conditions (qRT–PCR assay, normalized to β-Actin). **(F)** Expression levels of osteogenesis-related proteins (ALP, RUNX2, BMP2 and OCN) in PDLSCs under normal (Ctrl) or high glucose (HG) conditions (Western blot analysis, normalized to β-Actin). The data are presented as the mean ± SD (*n* ≥ 3). The *p* value was based on *t* test. ^**^ *p* < 0.01 and ^***^ *p* < 0.001 represent significant differences between the indicated columns, while *ns* represents no significant difference.

Furthermore, PDLSCs were incubated in either normal (referred the Ctrl group) or high glucose (referred the HG group) environments, and the osteogenic differentiation potentials of PDLSCs under different conditions were further evaluated. As shown by ALP staining and ALP activity measurement, fewer ALP staining-positive PDLSCs were found under high glucose conditions (**Figure 1A**), and PDLSCs in the HG group exhibited lower ALP activity than those in the Ctrl group (**Figure 1B**). The results of Alizarin Red staining also suggested that incubation of PDLSCs under high glucose conditions led to a decrease in the formation of mineralized nodules (**Figures 1C, D**). Furthermore, the expression levels of osteoblast differentiation-related genes (*ALP*, *RUNX2*, *BMP2* and *OCN*) (**Figure 1E**) and osteoblast differentiation-related proteins (ALP, BMP2 and OCN) (**Figure 1F**) were significantly decreased in PDLSCs under high glucose conditions.

### 3.2 PDLSCs incubated under high glucose conditions exhibited increased accumulation of ROS and NADPH during osteogenic differentiation process

**Figure 2:**
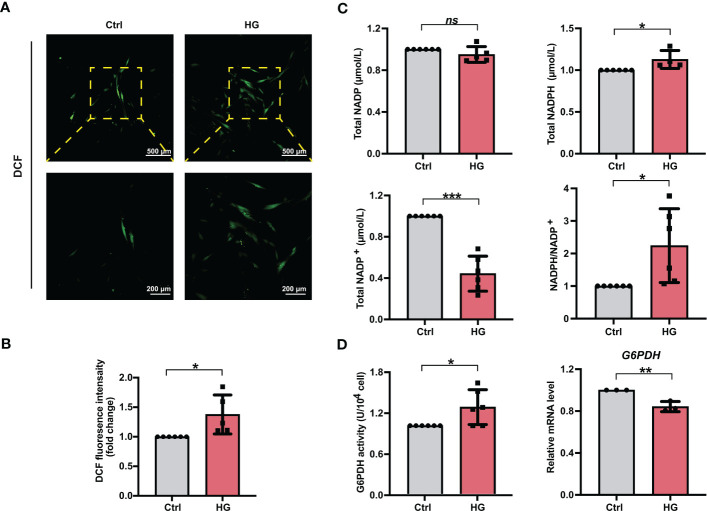
PDLSCs incubated under high glucose conditions exhibited increased ROS and NADPH accumulation during osteogenic induction. **(A)** Images of DCF fluorescence showing the general ROS levels in PDLSCs under normal (Ctrl) or high glucose (HG) conditions (scan bar = 500 μm in the above and 200 μm in the below). **(B)** Quantitative analysis of intracellular ROS levels measured by flow cytometry in the relative DCF fluorescence intensity. **(C)** Levels of NADP, NADPH and NADP^+^ and the ratio of NADP/NADP^+^ in PDLSCs under normal (Ctrl) or high glucose (HG) conditions. **(D)** G6PDH activity (left) and mRNA level (right) of G6PDH in PDLSCs under normal (Ctrl) or high glucose (HG) conditions. The data are presented as the mean ± SD (*n* ≥ 3). The *p* value was based on *t* test. ^*^ *p* < 0.05, ^**^ *p* < 0.01 and ^***^ *p* < 0.001 represent significant differences between the indicated columns, while *ns* represents no significant difference.

To investigate the effects of high glucose conditions on cellular ROS accumulation during osteogenic induction, we measured the ROS levels in different groups of PDLSCs. After the treatment of DCFH-DA, more fluorescent DCF were observed in PDLSCs under high glucose conditions (**Figure 2A**). Moreover, the relative fluorescence intensity of DCF in PDLSCs from the HG group was also significantly higher than that in cells from the Ctrl group (**Figure 2B**).

We next measured the total NADP (NADP^+^ + NADPH), NADPH, NADP^+^ levels as well as the NADPH/NADP^+^ ratio in PDLSCs under normal or high glucose conditions. Although high glucose incubation of PDLSCs did not change total NADP levels, PDLSCs incubated under high glucose conditions showed higher NADPH levels, lower NADP^+^ levels, and hence a significantly elevated NADPH/NADP^+^ ratio (**Figure 2C**). Furthermore, we detected the enzymatic activity and mRNA level of G6PDH (a crucial enzyme involved in the synthesis of NADPH) and found that although the mRNA level of G6PDH was lowered, PDLSCs in the HG group possessed a significant stronger enzymatic activity of cellular G6PDH (**Figure 2D**).

### 3.3 6-AN treatment or G6PDH downregulation reduced cellular ROS accumulation of PDLSCs under high glucose conditions

**Figure 3:**
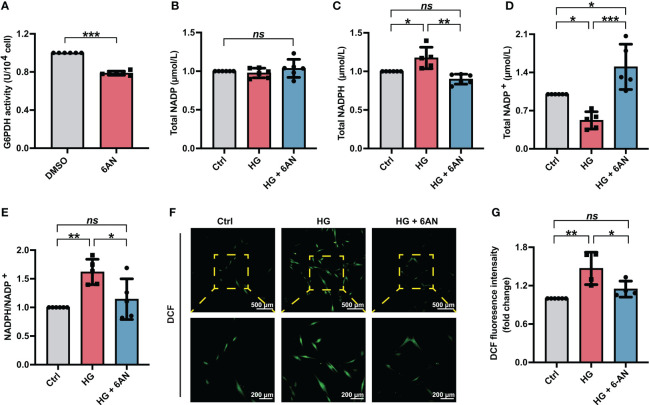
6-AN treatment reduced cellular ROS accumulation in PDLSCs under high glucose conditions. **(A)** G6PDH activity in PDLSCs with or without 6-AN treatment. **(B-E)** Levels of NADP, NADPH and NADP^+^ and the ratio of NADP/NADP^+^ in PDLSCs under normal (Ctrl) or high glucose conditions without (HG) or with 6-AN treatment (HG + 6-AN). **(F)** Images of DCF fluorescence showing the general ROS level in PDLSCs under normal (Ctrl) or high glucose conditions without (HG) or with 6-AN treatment (HG + 6-AN) (scan bar = 500 μm in the above and 200 μm in the below). **(G)** Quantitative analysis of intracellular ROS levels measured by flow cytometry in the relative DCF fluorescence intensity. The data are presented as the mean ± SD (*n* ≥ 4). The *p* value was based on *t* test (for two unpaired groups) and one-way analysis of variance (one-way ANOVA, for more than two groups). ^*^ *p* < 0.05, ^**^ *p* < 0.01 and ^***^ *p* < 0.001 represent significant differences between the indicated columns, while *ns* represents no significant difference.

To further clarify the role of NADPH in high glucose-induced ROS accumulation, we first treated PDLSCs with the G6PDH pharmacological inhibitor 6-AN. The inhibitory effect of 6-AN on the enzymatic activity of G6PDH were confirmed (**Figure 3A**). We found that in high glucose-incubated PDLSCs, 6-AN treatment significantly decreased the NADPH levels (**Figure 3C**) and NADPH/NADP^+^ ratio (**Figure 3E**) and increased the levels of NADP^+^ (**Figure 3D**). The total NADP levels were not altered (**Figure 3B**). Furthermore, after 6-AN treatment, the number of fluorescent DCF-positive cells (**Figure 3F**) and the relative fluorescence intensity of DCF (**Figure 3G**) was significantly decreased in PDLSCs under high glucose conditions.

**Figure 4:**
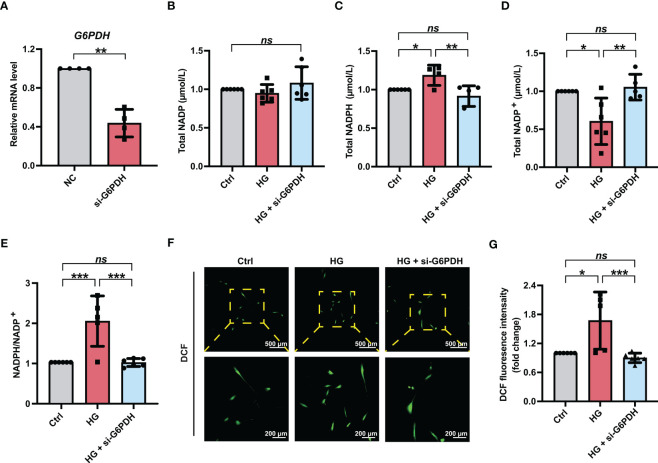
Downregulation of G6PDH expression reduced cellular ROS accumulation in PDLSCs under high glucose conditions. **(A)** mRNA level of G6PDH in PDLSCs with or without si-G6PDH treatment (qRT–PCR assay, normalized to β-Actin). **(B-E)** Levels of NADP, NADPH and NADP^+^ and the ratio of NADP/NADP^+^ in PDLSCs under normal (Ctrl) or high glucose conditions without (HG) or with si-G6PDH treatment (HG + si-G6PDH). **(F)** Images of DCF fluorescence showing the general ROS level in PDLSCs under normal (Ctrl) or high glucose conditions without (HG) or with si-G6PDH treatment (HG + si-G6PDH) (scan bar = 500 μm in the above and 200 μm in the below). **(G)** Quantitative analysis of intracellular ROS levels measured by flow cytometry in the relative DCF fluorescence intensity. The data are presented as the mean ± SD (*n* ≥ 4). The *p* value was based on *t* test (for two unpaired groups) and one-way analysis of variance (one-way ANOVA, for more than two groups). ^*^ *p* < 0.05, ^**^ *p* < 0.01 and ^***^ *p* < 0.001 represent significant differences between the indicated columns, while *ns* represents no significant difference.

Moreover, to obtain a more selective blockade of G6PDH activity, PDLSCs were treated with siRNA targeting G6PDH (si-G6PDH). The inhibitory effect of si-G6PDH on the mRNA level of G6PDH were confirmed (**Figure 4A**). Similar to the PDLSCs with 6-AN treatment, transfection with si-G6PDH led to a significant decrease in the NADPH levels (**Figure 4C**) and NADPH/NADP^+^ ratio (**Figure 4E**) and an increase in the levels of NADP^+^ (**Figure 4D**), with the total NADP levels remaining unchanged (**Figure 4B**). In addition, the number of fluorescent DCF-positive cells (**Figure 4F**) and the relative fluorescence intensity of DCF (**Figure 4G**) in PDLSCs under high glucose conditions was significantly reduced after si-G6PDH transfection.

### 3.4 6-AN treatment alleviated the high glucose-induced impairment of PDLSC osteogenic differentiation

Given that suppressing NADPH production (by 6-AN treatment or G6PDH downregulation) can significantly alleviate ROS accumulation in PDLSCs under high glucose conditions, we hypothesized that NADPH might contribute to the high glucose-induced impairment of PDLSC osteogenic differentiation. Here, we first treated PDLSCs with exogenous NADPH (from 0-500 nmol/L) under normal glucose conditions to determine the role of NADPH on cellular osteogenic differentiation. We found that the number of ALP staining-positive cells and cellular ALP activity were significantly decreased after NADPH treatment, with no significant difference in the effects among NADPH concentrations (**Figures S2A, B**). In addition, exogenous NADPH administration dramatically reduced the formation of mineralized nodules, and this effect became more pronounced with increasing NADPH concentration (**Figures S2C, D**).

**Figure 5:**
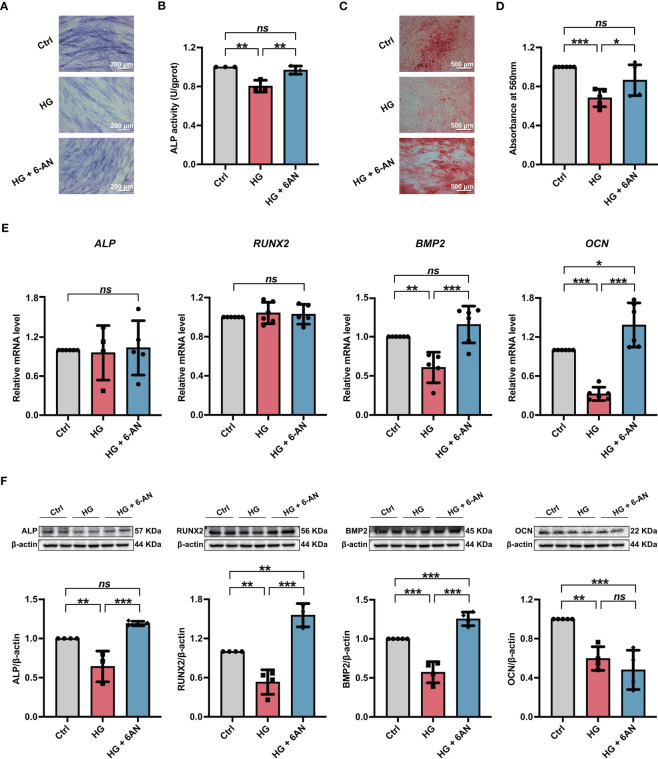
Modulation of NADPH generation *via* 6-AN treatment ameliorated the high glucose-induced impairment of PDLSC osteogenic differentiation. **(A)** ALP staining of PDLSCs under normal (Ctrl) or high glucose conditions without (HG) or with 6-AN treatment (HG + 6-AN) following a 7-day osteogenic induction (scan bar = 200 μm). **(B)** ALP activity assay of PDLSCs under normal (Ctrl) or high glucose conditions without (HG) or with 6-AN treatment (HG + 6-AN) following a 7-day osteogenic induction. **(C)** Alizarin Red staining of PDLSCs under normal (Ctrl) or high glucose conditions without (HG) or with 6-AN treatment (HG + 6-AN) following a 21-day osteogenic induction (scan bar = 500 μm). **(D)** Quantitative analysis of mineralized nodules formed by PDLSCs under normal (Ctrl) or high glucose conditions without (HG) or with 6-AN treatment (HG + 6-AN) following a 21-day osteogenic induction. **(E)** Expression levels of osteogenesis-related genes (*ALP*, *RUNX2*, *BMP2* and *OCN*) in PDLSCs under normal (Ctrl) or high glucose conditions without (HG) or with 6-AN treatment (HG + 6-AN) (qRT–PCR assay, normalized to β-Actin). **(F)** Expression levels of osteogenesis-related proteins (ALP, RUNX2, BMP2 and OCN) in PDLSCs under normal (Ctrl) or high glucose conditions without (HG) or with 6-AN treatment (HG + 6-AN) (Western blot analysis, normalized to β-Actin). The data are presented as the mean ± SD (*n* ≥ 3). The *p* value was based on one-way analysis of variance (one-way ANOVA). ^*^ *p* < 0.05, ^**^ *p* < 0.01 and ^***^ *p* < 0.001 represent significant differences between the indicated columns, while *ns* represents no significant difference.

We further investigated the influences of inhibiting NADPH generation on the osteogenic differentiation potential of PDLSCs under high glucose conditions. Here, we found that 6-AN treatment significantly increased cellular ALP activity (**Figures 5A, B**) and the mineralized nodules formation of PDLSCs under high glucose conditions (**Figures 5C, D**). In addition, although no significant differences were found in *ALP* and *RUNX2* (osteoblast differentiation-related genes) levels, the expression levels of *BMP2* and *OCN* were significantly increased in PDLSCs after 6-AN treatment (HG + 6-AN group) (**Figure 5E**). Similarly, the results of western blot analysis also suggested that 6-AN treatment markedly enhanced the expression levels of osteoblast differentiation-related proteins (ALP, RUNX2 and BMP2) in PDLSCs under high glucose conditions, with no significant change observed in the expression level of OCN (**Figure 5F**).

### 3.5 G6PDH downregulation alleviated the high glucose-induced impairment of PDLSC osteogenic differentiation

**Figure 6:**
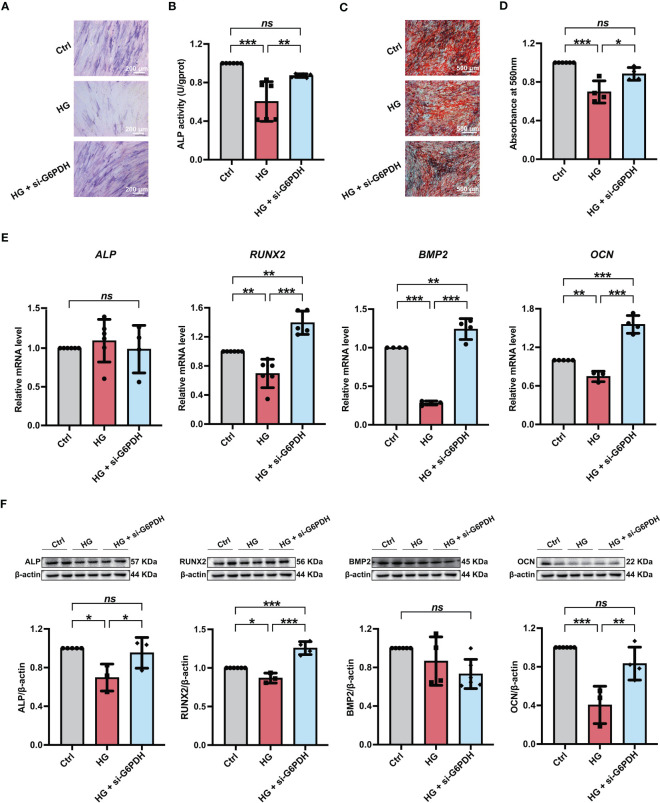
Modulation of NADPH generation *via* downregulation of G6PDH expression ameliorated the high glucose-induced impairment of PDLSC osteogenic differentiation. **(A)** ALP staining of PDLSCs under normal (Ctrl) or high glucose conditions without (HG) or with si-G6PDH treatment (HG + si-G6PDH) following a 7-day osteogenic induction (scan bar = 200 μm). **(B)** ALP activity assay of PDLSCs under normal (Ctrl) or high glucose conditions without (HG) or with si-G6PDH treatment (HG + si-G6PDH) following a 7-day osteogenic induction. **(C)** Alizarin Red staining of PDLSCs under normal (Ctrl) or high glucose conditions without (HG) or with 6-AN treatment (HG + si-G6PDH) following a 21-day osteogenic induction (scan bar = 500 μm). **(D)** Quantitative analysis of mineralized nodules formed by PDLSCs under normal (Ctrl) or high glucose conditions without (HG) or with 6-AN treatment (HG + si-G6PDH) following a 21-day osteogenic induction. **(E)** Expression levels of osteogenesis-related genes (*ALP*, *RUNX2*, *BMP2* and *OCN*) in PDLSCs under normal (Ctrl) or high glucose conditions without (HG) or with 6-AN treatment (HG + si-G6PDH) (qRT–PCR assay, normalized to β-Actin). **(F)** Expression levels of osteogenesis-related proteins (ALP, RUNX2, BMP2 and OCN) in PDLSCs under normal (Ctrl) or high glucose conditions without (HG) or with 6-AN treatment (HG + si-G6PDH) (Western blot analysis, normalized to β-Actin). The data are presented as the mean ± SD (*n* ≥ 4). The *p* value was based on one-way analysis of variance (one-way ANOVA). ^*^ *p* < 0.05, ^**^ *p* < 0.01 and ^***^ *p* < 0.001 represent significant differences between the indicated columns, while *ns* represents no significant difference.

Furthermore, we used si-G6PDH transfection to suppress NADPH generation in PDLSCs under high glucose conditions and measured its influences on cellular osteogenic differentiation. After si-G6PDH transfection, a significant increase of ALP staining-positive cells (**Figure 6A**) and cellular ALP activity (**Figure 6B**) was observed in PDLSCs under high glucose conditions. Moreover, after si-G6PDH transfection, PDLSCs under high glucose conditions exhibited an increased number of mineralized nodules (**Figures 6C, D**), as evidenced by Alizarin Red staining. In addition, si-G6PDH transfection also significantly enhanced the expression level of *RUNX2*, *BMP2* and *OCN* (osteoblast differentiation-related genes), while the *ALP* level was not changed (**Figure 6E**). Data from western blot analysis also showed a significant increase in the expression levels of osteoblast differentiation-related proteins (ALP, RUNX2 and OCN) after transfection with si-G6PDH, with no significant change in the protein level of BMP2 (**Figure 6F**).

## 4 Discussion

Substantial studies have demonstrated that Diabetes mellitus is one of the common risk factors for periodontal disease, and the chronic hyperglycemic environment might be the major cause of diabetes-related pathogenesis (3, 41). Moreover, high glucose-induced cell dysfunction, such as impaired osteogenic differentiation potential, has been considered as one of the most significant challenges in stem cell-based (especially PDLSC-based) periodontal therapies for diabetic patients (6, 42–44). Indeed, PDLSCs are promising candidates for periodontal regeneration due to their advantage in repairing alveolar bone defects and mediating periodontal tissue regeneration (10, 45). However, given that the mechanisms underlying the high glucose-induced impairment of cell differentiation remain poorly understood, the application of PDLSCs to promote periodontal tissue regeneration in patients with diabetes is still in its infancy. Recently, increasing evidence has suggested that ROS play a vital role in the regulation of cell differentiation (17, 18). Many studies also found that cells incubated under high glucose conditions showed dysregulation of intracellular ROS and NADPH, indicating that they might contribute to high glucose-induced cellular osteogenic differentiation impairment (29, 36). However, the exact role of ROS and NADPH in the impairment of PDLSC osteogenic differentiation under high glucose conditions remains unexplored. Therefore, this study mainly aimed to reveal the underlying mechanisms by which high glucose conditions compromise the osteogenic differentiation of PDLSCs and identify how the regulation of intracellular ROS and NADPH levels correlates with cell osteogenesis under high glucose conditions.

According to reported studies and our previous protocols (13, 46, 47), PDLSCs in the present study were incubated in medium with either 5 mmol/L glucose, which was chosen to simulate normal conditions *in vitro* (referred as the Ctrl group), or 25 mmol/L glucose, which was used to simulate high glucose conditions (referred as the HG group). We found an adverse effect of high glucose on PDLSC osteogenic differentiation in this study, as evidenced by a significant decrease in cellular ALP activity, formation of mineralized nodules, and expression levels of osteogenesis-related proteins and genes (**Figure 1**). These results were consistent with reported studies and our previous findings that incubation under high glucose can lead to severe cell dysfunction, including impaired osteogenic differentiation and enhanced apoptosis (13, 29), and further indicated the necessity to explore the mechanisms underlying high glucose-compromised osteogenic differentiation of PDLSCs.

Reactive oxygen species (ROS) are oxygen-derived small molecules that play a vital role in regulating cell differentiation (48). Many studies have also demonstrated that increased intracellular ROS levels are also the important cause of diabetes-related cell damage (19). To investigate the exact role of ROS in PDLSC osteogenic differentiation, the intracellular ROS levels in PDLSCs incubated under high glucose conditions were measured. We found that during osteogenic induction, more DCF staining-positive cells and higher relative fluorescence intensity of DCF in cells were observed in the HG group than in the Ctrl group (**Figures 2A, B**), indicating increased intracellular ROS levels in PDLSCs incubated under high glucose conditions. Our results were consistent with studies which showed that high glucose-induced excessive ROS accumulation is responsible for impaired cellular differentiation potential (17, 29). Generally, excessive ROS have long been considered as a main contributor to the oxidation of intracellular components and subsequent cellular dysfunction, and the increase in ROS can block cell osteogenic differentiation but is required for the process of adipocyte differentiation (26, 27). A study of Tan et al. revealed that the expression of osteogenesis-related proteins and genes observed was decreased in stem cells from older volunteers and this decrease might be the result of increased intracellular levels of ROS (49). Moreover, Lee et al. used exogenous H_2_O_2_ to construct an *in vitro* high ROS environment and found that exogenous H_2_O_2_ administration could significantly reduce cellular ALP activity, an important osteogenesis-related marker, and lead to impairment of cellular osteogenic differentiation (50). Hence, together with previous studies, our present study suggested that increased intracellular ROS levels might be responsible for impaired osteogenic differentiation under high glucose conditions, which also demonstrated a considerable need to explore new effective strategies specifically targeting intracellular ROS levels to combat high glucose-induced cell dysfunction.

Nicotinamide adenine dinucleotide phosphate (NADPH) is well known as a critical ROS scavenger of cellular antioxidant systems. But whether it is involved in the high glucose-induced increased ROS accumulation is still unclear. Hence, we further explored the role of NADPH in the high glucose-induced excessive ROS generation in PDLSCs. We first measured the total NADP, NADPH and NADP^+^ levels as well as calculated the NADPH/NADP^+^ ratio in PDLSCs under high glucose conditions. Interestingly, although no significant change was found in total NADP levels, PDLSCs incubated under high glucose conditions exhibited a significant reduction in NADP^+^ levels and an increase in NADPH levels and the NADPH/NADP^+^ ratio (**Figure 2C**), indicating an increased NADPH production in cells under high glucose conditions. These results were consistent with previous studies suggesting that glucose is a main source of NADPH production and increasing glucose concentration can acutely promote NADPH generation from NADP^+^ and increase intracellular NADPH levels (51, 52). Ying et al. also revealed that deprivation of glucose can lead to a great reduction in the generation of NADPH (53). Moreover, increased NADPH production has been associated with some diabetic complications. Wei et al. demonstrated that podocytes isolated from diabetic rats showed increased NADPH levels, and inhibiting NADPH production could help to combat diabetic-caused impairment of podocytes (36). However, some studies have also suggested that exposure to high glucose conditions may reduce NADPH supply and decrease the NADPH/NADP^+^ ratio in endothelial cells, thereby impairing cellular resistance to oxidative stress (54). These controversial findings may be due to the variation in glucose tolerance in different cell lineages, which can further change the activity of NADPH-related enzymes. For example, although endothelial cells exhibited lowered NADPH production under high glucose conditions (54), an increase in NADPH levels was found in cancer calls and islet β-cells when incubated in medium with elevated glucose concentrations (52, 53). Taken together, our findings suggested that incubation under high glucose conditions can lead to increased NADPH production in PDLSCs.

Given that glucose-6-phophate dehydrogenase (G6PDH) is an essential enzyme in the synthesis and function of NADPH (55, 56), we inhibited cellular G6PDH activity with the pharmacological inhibitor 6-AN (100 nmol/L) (57) or siRNA targeting G6PDH (si-G6PDH) to reduce NADPH generation. In the current study, we found that PDLSCs treated with either 6-AN or si-G6PDH exhibited decreased NAPDH levels and NADPH/NADP^+^ ratio, accompanied by a significant decrease in intracellular ROS levels (**Figures 3**, **4**). These results indicated that increased NADPH production may be responsible for high glucose-induced excessive ROS accumulation in PDLSCs. Consistently, substantial evidence has suggested that in the case of a high glucose environment, increased ROS levels can not only be a direct result of sorbitol accumulation but also an indirect result of reactivation of NADPH oxidases (NOXs) *via* NADPH consumption (58). In fact, in addition to its role as an ROS scavenger, NADPH can also contribute to ROS generation by NOXs (31). For example, in a case of chronic granulomatous disease, NADPH can serve as an electron donor, and the electrons are transferred from NADPH to flavin adenine dinucleotide (FAD) and then be used to activate NOX2. And this oxidase can further generate superoxide by transferring an electron from NADPH in the cytosol to oxygen on the extracellular space, leading to increased ROS generation (59, 60). However, other studies found that increased NADPH generation *via* G6PDH overexpression can help to lower ROS accumulation-induced apoptosis and mitochondrial damage in cochlear cells, and further combat age-related hearing loss (61). A study by Cano et al. also demonstrated that the ROS-induced epithelial cells death could be exaggerated by inhibiting NADPH supply (62). Moreover, other studies also reported that high glucose-induced excessive generation of ROS might be associated with dysregulation of glutathione peroxidase and mitochondrial function (30, 63). Therefore, more in-depth studies are needed to elucidate the different effects of NADPH on ROS generation and other pathways involved in high glucose-induced ROS generation.

NADPH is also an essential regulator in several important synthesis reactions, such as the production of fatty acids and nucleotides, to maintain cell growth (64). We treated PDLSCs with NADPH (from 0-500 nmol/L) under normal glucose conditions to explore whether NADPH is involved in cellular osteogenic differentiation (65). The results suggested that administration of exogenous NADPH can dramatically reduce the osteogenic differentiation of PDLSCs, as evidenced by a significant decrease in ALP staining-positive cells, cellular ALP activity and the formation of mineralized nodules (**Figure S2**). In addition, the inhibitory effect of NADPH on PDLSC osteogenic differentiation became more pronounced with increasing NADPH concentrations. Furthermore, to investigate the role of NADPH in the high glucose-induced impairment of PDLSC osteogenic differentiation, PDLSCs were further treated with either 6-AN or si-G6PDH, and the osteogenic differentiation was evaluated. We found that cells treated with either 6-AN or si-G6PDH exhibited higher ALP activity and formation of mineralized nodules, as well as increased expression levels of osteoblast differentiation-related genes and proteins (**Figures 5**, **6**), indicating an enhancement in cellular osteogenic differentiation after inhibiting NADPH production. Consistently, Wei et al. found that increased NADPH levels are a critical cause of mitochondrial malfunction in glomeruli isolated from diabetic rats, and that mitochondrial function was further improved by reducing NADPH levels with Roux-en-Y Gastric Bypass (RYGB) surgery. The inhibitory effects of RYGB surgery on high glucose-induced damage can be significantly blocked after administration of NADPH (36). In addition, Zhu et al. found that although 20-500 nmol/L of NADPH supplement ameliorated mitochondrial dysfunction and protected cells against myocardial ischemia/reperfusion (I/R) injury, its protective effect was weakened as the concentration increased (65). Therefore, consistent with previous studies, our current findings suggested that high glucose-induced excessive NADPH production exerted an adverse effect on the osteogenic differentiation of PDLSCs. To our knowledge, this is the first report that NADPH plays an essential role in cell function, particularly osteogenic differentiation.

**Figure 7:**
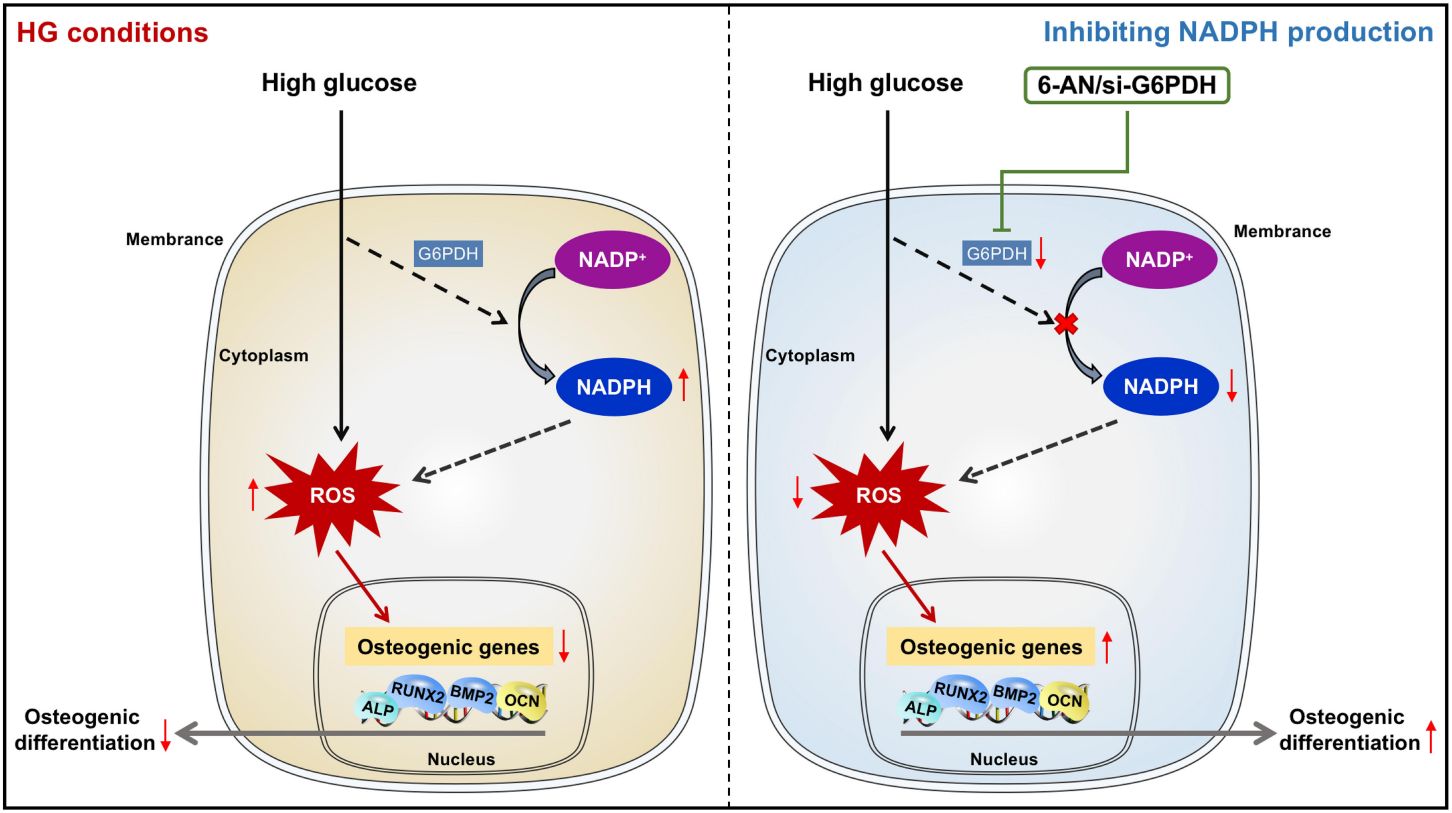
Diagram showing that NADPH-dependent ROS accumulation plays an essential role in the impaired osteogenic differentiation of PDLSCs under high glucose conditions.

Despite recent advances in stem cell biology, the application of stem cell therapy in periodontal treatment of diabetic patients remains in its infancy, largely due to the dysregulation of the reparative cell population exposed to diabetic cues, especially high glucose conditions. We found in the present study that impaired osteogenic differentiation of PDLSCs incubated under high glucose conditions occurred with increased ROS and NADPH generation. Moreover, inhibiting NADPH production and downstream ROS accumulation could reverse the impairment of PDLSC osteogenic differentiation induced by high glucose incubation. Our current data revealed for the first time that impaired osteogenic differentiation of PDLSCs under high glucose conditions occurs with an increase in NADPH-dependent ROS accumulation (**Figure 7**). These findings may provide new insights into the cellular and molecular events that contribute to high glucose-regulated cell differentiation and identify a new therapeutic target for promoting multiple tissue regeneration in diabetic patients.

## Data availability statement

The original contributions presented in the study are included in the article/**Supplementary Material**. Further inquiries can be directed to the corresponding authors.

## Ethics statement

The studies involving human participants were reviewed and approved by the Ethics Committee of the Stomatological Hospital of FMMU (201203). The patients/participants provided their written informed consent to participate in this study.

## Author contributions

Y-LZ and YA contributed to the conception and design of the study. Y-LZ, YA, L-JS and H-LQ did the job of acquisition and analysis. XL, X-TH and R-XW did the job of interpretation of data and manuscript writing. F-MC, B-MT and YY contributed to the study conception and design, financial support and manuscript writing. All authors read and approved the final manuscript.

## Supplementary material

Supplementary Figure 1 Isolation and characterization of PDLSCs. **(A)** Primary cells migrated from PDL tissues observed on day 7 (above) and day 14 (below) (scale bar = 500 μm). **(B)** Colony formation ability of PDLSCs: colonies in a macroscopic view and a single colony observed microscopically (scale bar = 500 µm). **(C)** Proliferative activity of PDLSCs assessed by the CCK-8 assay during an 8-day culture. **(D)** Alizarin Red staining (left; scale bar = 500 µm), Oil Red O staining (middle; scale bar = 200 µm) and Alcian Blue staining (right; scale bar = 500 µm) of the PDLSCs following a 21-day osteogenic, adipogenic or chondrogenic induction. **(E)** Surface markers of PDLSCs assessed by flow cytometry analysis.

Supplementary Figure 2 Administration of NADPH led to impaired osteogenic differentiation of PDLSCs. **(A)** ALP staining of PDLSCs treated with different concentrations of NADPH under normal glucose conditions (scan bar = 200 μm). **(B)** ALP activity assay of PDLSCs treated with different concentrations of NADPH under normal glucose conditions. **(C)** Alizarin Red staining of PDLSCs treated with different concentrations of NADPH under normal glucose conditions (scan bar = 500 μm). **(D)** Quantitative analysis of mineralized nodules formed by PDLSCs treated with different concentrations of NADPH under normal glucose conditions. The data are presented as the mean ± SD (*n* ≥ 3). The *p* value was based on one-way analysis of variance (one-way ANOVA). ^*^ *p* < 0.05, ^**^ *p* < 0.01 and ^***^ *p* < 0.001 represent significant differences between the indicated columns, while *ns* represents no significant difference.
