# Supplementary figures and images for: NADPH-dependent ROS accumulation contributes to the impaired osteogenic differentiation of periodontal ligament stem cells under high glucose conditions
Source: Front Endocrinol (Lausanne). 2023 Jun 7;14:1152845. doi: 10.3389/fendo.2023.1152845 (PMC10282952; doi:10.3389/fendo.2023.1152845)

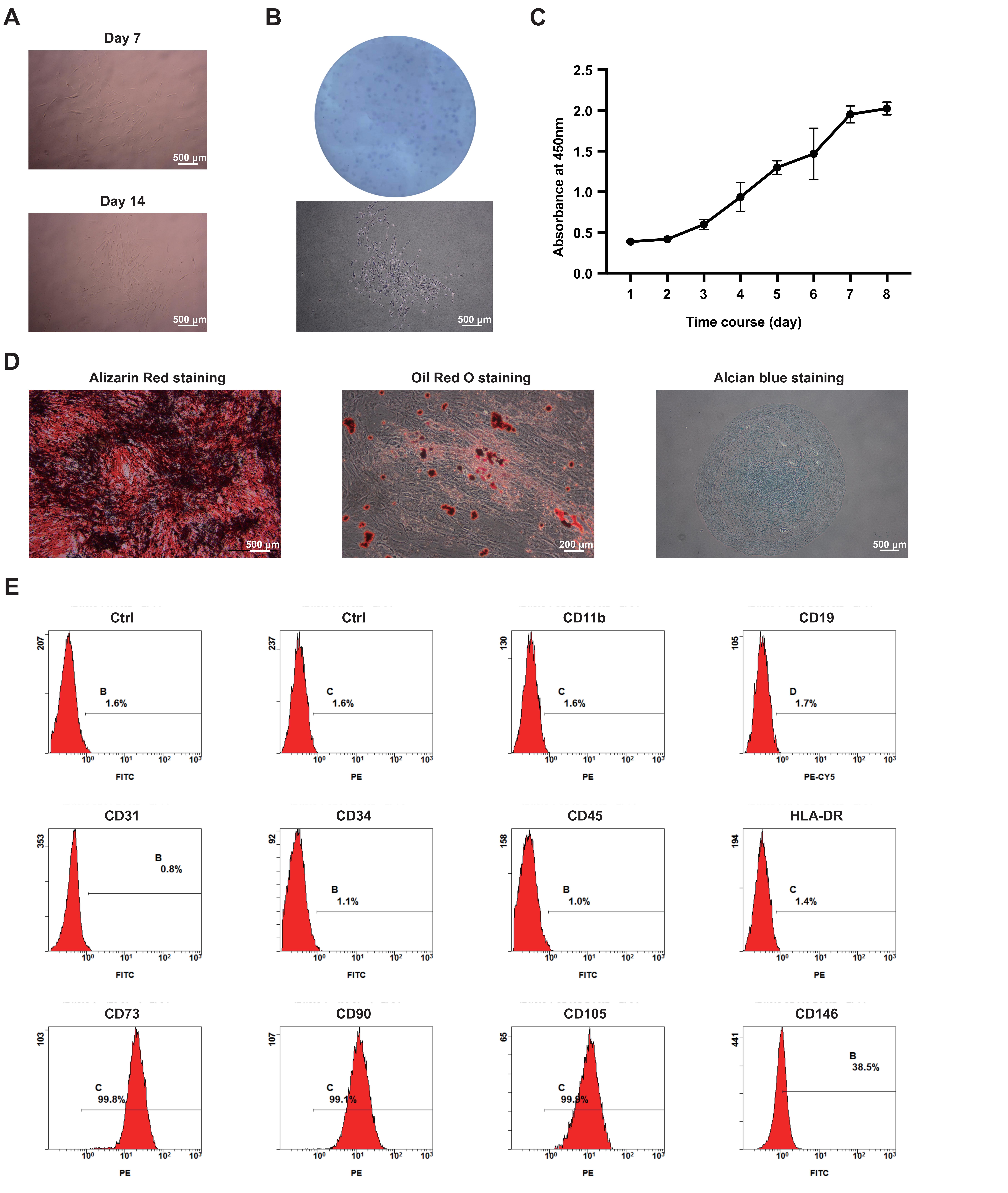

Supplement: Supplementary Figure 1 — Isolation and characterization of PDLSCs. (A) Primary cells migrated from PDL tissues observed on day 7 (above) and day 14 (below) (scale bar = 500 μm). (B) Colony formation ability of PDLSCs: colonies in a macroscopic view and a single colony observed microscopically (scale bar = 500 µm). (C) Proliferative activity of PDLSCs assessed by the CCK-8 assay during an 8-day culture. (D) Alizarin Red staining (left; scale bar = 500 µm), Oil Red O staining (middle; scale bar = 200 µm) and Alcian Blue staining (right; scale bar = 500 µm) of the PDLSCs following a 21-day osteogenic, adipogenic or chondrogenic induction. (E) Surface markers of PDLSCs assessed by flow cytometry analysis. [file Image_1.jpeg]

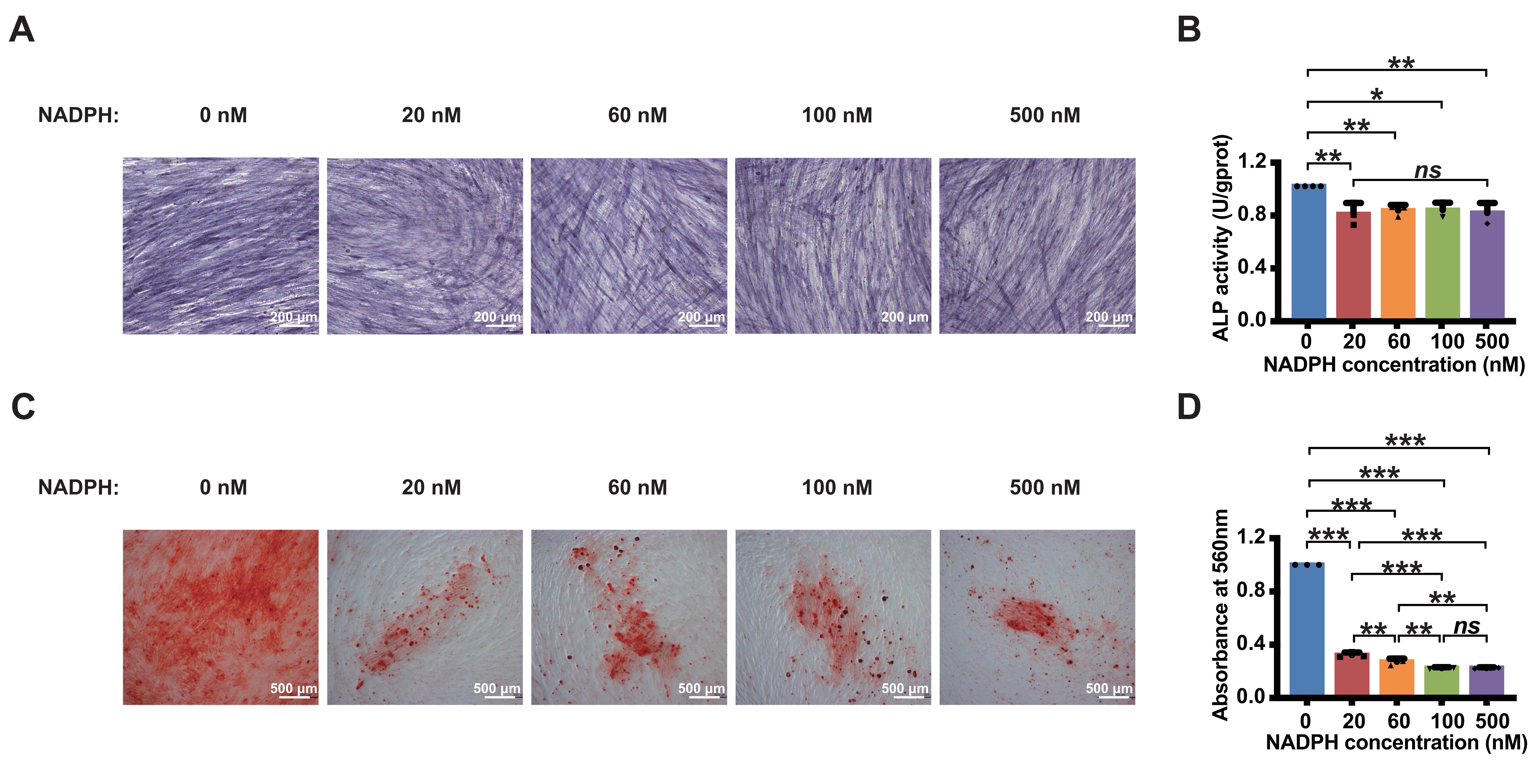

Supplement: Supplementary Figure 2 — Administration of NADPH led to impaired osteogenic differentiation of PDLSCs. (A) ALP staining of PDLSCs treated with different concentrations of NADPH under normal glucose conditions (scan bar = 200 μm). (B) ALP activity assay of PDLSCs treated with different concentrations of NADPH under normal glucose conditions. (C) Alizarin Red staining of PDLSCs treated with different concentrations of NADPH under normal glucose conditions (scan bar = 500 μm). (D) Quantitative analysis of mineralized nodules formed by PDLSCs treated with different concentrations of NADPH under normal glucose conditions. The data are presented as the mean ± SD (n ≥ 3). The p value was based on one-way analysis of variance (one-way ANOVA). * p < 0.05, ** p < 0.01 and *** p < 0.001 represent significant differences between the indicated columns, while ns represents no significant difference. [file Image_2.jpeg]
